# Border Environmental Justice PPGIS: Community-Based Mapping and Public Participation in Eastern Tijuana, México

**DOI:** 10.3390/ijerph18031349

**Published:** 2021-02-02

**Authors:** Carolina Prado, Colectivo Salud y Justicia Ambiental, Red de Ciudadanos para el Mejoramiento de las Comunidades

**Affiliations:** 1 Department of Environmental Studies, San José State University, 1 Washington Sq., San José, CA 95192, USA; 2 Colectivo Salud y Justicia Ambiental, AC, Avenida del Fuerte #15861 Colonia Campestre Murúa, Tijuana 22455, Mexico; anibalm@environmentalhealth.org; 3 Red de Ciudadanos para el Mejoramiento de las Comunidades, Mexicanidad #3030, Colonia 10 de Mayo, Tijuana 22476, Mexico; jorge.recimec@hotmail.com

**Keywords:** community based mapping, environmental justice, urban planning, public participation

## Abstract

Community mapping projects have been studied as important contributions to the field of environmental justice and Public Participation Geographic Information Systems (PPGIS). As a collaborative project between the Colectivo Salud y Justicia Ambiental and Red de Ciudadanos por el Mejoramiento de las Comunidades (RECIMEC), the “Mapeo Comunitario de la Zona Alamar” was created as a mechanism for community participation in the urban planning process in Tijuana, México. This paper outlines the project’s community mapping process, including planning, data collection, priority identification, and data submission. Results from this community mapping project are analyzed including the (1) particular environmental risks and goods in this border region, (2) the influence that the project data had on the urban planning process, and (3) the impact that the community mapping process had on community organizing capacity. Our findings point to particular environmental challenges in this border city including clandestine trash dumps, and contaminated water runoff points. The mapping project influenced the land use planning process by identifying the key environmental risks and goods to prioritize in the zoning and ground truthing urban planning data. The community mapping project also had a key impact on community organizing through the fomenting of knowledge and relationships between community members and government representatives at the city’s urban planning agency.

## 1. Introduction

In the U.S., environmental justice was established as a term to explain the links between the siting of environmental ills, or land uses that deteriorate a person’s health and quality of life (such as garbage dumps), and the racial and class compositions of the neighborhoods that were chosen [1,2]. Changes in global production systems have reshaped the terrain of environmental justice by increasing the distance between the communities affected and the actors responsible for environmental degradation [3]. Though there is a theoretical understanding of how local environmental justice is shaped by global dynamics, research in this field is predominantly in non-border regions of the many countries around the world experiencing environmental inequality [4,5]. While studies of global environmental inequalities across populations exist generally [6,7,8], few deal with the unique conditions presented in the many urban border regions that exist globally. These borders experience environmental injustice, both between neighboring countries and within each country that are distinct from non-border regions [9]. This border environmental justice includes distributive inequality, or unequal distribution of environmental ills and goods [10], procedural inequality, or lack of participation of marginalized communities in environmental decision-making [11], and recognition injustice, or the exclusion of certain residents from the role of stakeholder [12]. Environmental justice in the border region is shaped by the social, economic, and environmental cohesion across the boundary, the core-periphery relationship between the two countries and the region’s fragmented environmental governance [9]. While the foundational elements have been identified, there is a gap in understanding of the types of land uses that influence environmental injustice in the border region. When it comes to environmental injustices, land use has long been a focus of research on the relationship between the built environment and health. The first objective of this research is to answer the question: what are the localized environmental risks to health and environmental goods identified by border residents? 

Within environmental justice research, mapping has been helpful to identify environmental hazards [13,14,15]. The field of mapping has expanded to include a critical analysis of the ways historically top-down technologies like Geographic Information Systems (GIS) can exclude community knowledge. As a response, the field of Public Participation GIS (PPGIS) has expanded the practice of mapping to include “spatially explicit methods and technologies for capturing and using spatial information in participatory planning processes” [16] (p. 1). PPGIS has been identified as a way for community members to facilitate community members’ participation in identifying favorable land use decisions and development preferences [17,18,19,20]. A key piece of community mapping in the PPGIS process is that community members use their local knowledge to inform valuation and understanding of particular places and the conditions they influence [21,22]. Environmental justice community mapping has involved engaging local residents in identifying the parameters of different urban environmental indicators like access to blue space [23], and cases of water injustice [24]. Two key debates within the field of PPGIS is the impact that community mapping processes have on land use planning processes and on community organizing capacities to improve environmental health conditions. This research engages this critical dynamic by analyzing the role that a community-based mapping project had on the urban planning process and community organizing in Eastern Tijuana, México. 

Typical of many border regions, environmental issues in Tijuana emerge from a confluence of rapid development, irregular infrastructure, and enforcement of environmental policy. When it comes to the industrial activities in the U.S.-Mexico border region, the cities of Tijuana and Ciudad Juárez have consistently been prioritized for the maquiladora industry siting. While in 2003, the number of maquiladoras in Tijuana was at 545, in 2012, there were only 223 maquiladoras in the city [25]. Due to the infrastructure and resource needs of these facilities, such as large inputs of water and outputs of wastewater, Tijuana also sees other pollution burdens like soil contamination [26], drought and lack of access to water [27], water pollution [28], air pollution [29,30], and illegal waste dumping [31,32]. 

**Figure 1:**
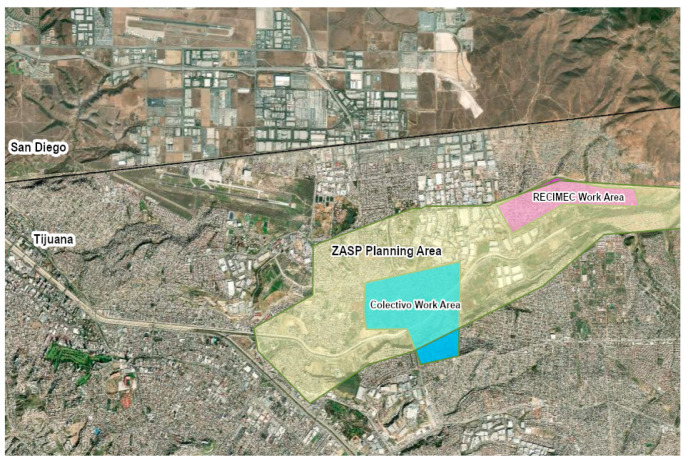
Zona Alamar Specific Plan (ZASP) Planning Area and Organization Work Areas.

The environmental justice and health risks in Tijuana urban communities are addressed by two organizations, the Colectivo Salud y Justicia Ambiental (formerly the Colectivo Chilpancingo) and RECIMEC (Red de Ciudadanos por el Mejoramiento de Las Comunidades), who used the tool of community mapping to address some of these issues. In 2015, the Colectivo’s campaign director learned that Tijuana’s urban planning agency (IMPLAN) was going to update the Zona Alamar Specific Plan (ZASP). This community-specific urban plan was to delineate the land use priorities and projects for the next ten years. The Colectivo decided to create a community mapping project in 2015 to add their priorities to the ZASP. A community mapping approach was chosen because the community-specific urban plan draft indicated to the Colectivo the importance of geo-referenced data for aligning appropriate land use zoning. This draft also illuminated the errors present in IMPLAN’s original data on environmental issues and land use. In 2017, RECIMEC joined the community mapping project in order to engage a larger set of communities within the ZASP planning area. The ZASP Planning area and the two organization’s work area are represented in the ZASP map in Figure 1. While the entire Zona Alamar delimited by the IMPLAN includes a polygon that is about 122 square kilometers, the seven colonias surveyed cover about 11 square kilometers of this area. This region was chosen as it encompasses the work areas for these two organizations. 

This paper outlines the key areas of this mapping project through an analysis of three key research questions. First, what are the key environmental risks and goods in the Zona Alamar? The second research question seeks to evaluate the influence of the community mapping project on the planning process for the ZASP. Finally, what are the mapping project’s impacts on community organizing capacity in the Colectivo and RECIMEC? The following sections outline the relevant literature, and methods used to identify priorities and collect data for the community mapping project. Then, project’s findings and implications are presented.

## 2. Literature Review

Land use planning is the process by which institutional bodies make decisions around the use of land in particular regions, like cities or counties. This land use planning influences both the distribution of environmental risks and goods (distributive justice) and the participation of diverse communities in environmental decision making (procedural justice). Land use planning has impacted distributive injustice in urban regions through practices like zoning. This practice is used by urban planners to delineate the types of uses (i.e., residential, commercial, industrial) that are legally allowed in different areas of the city. Zoning has historically created conditions where disadvantaged communities have less access to environmental goods like parks or walkable sidewalks and more exposure to environmental risks like freeways and toxic sites [16]. For example, in the early twentieth century, Phoenix Arizona had a progressive zoning plan that still fragmented land uses in the city that ultimately led to having less amenities in poor communities of color [33]. Urban planning scholars have made important distinctions between “proactive zoning,” where unwanted land uses like polluting firms are kept within specific zones, while “reactive” zoning creates buffer zones around unwanted land uses [34]. Proactive zoning is seen as more effective at protecting against environmental injustices. Other urban planning strategies geared toward improving environmental injustices include air quality plans, noise production measures, urban green infrastructure, and changes in housing infrastructure [35,36].

The element of distributive environmental justice, or the distribution of environmental risks and goods, is closely tied to land use. Built environment characteristics can have serious impacts on health outcomes for community members [37]. For example, exposure to the environmental risk of air pollution from nearby freeways can cause respiratory and cardiovascular disease [38], whereas having access to environmental goods like green space and parks has myriad health benefits like opportunities to do physical activity, improved mental health, and stress reduction [39,40,41]. The number and quality of parks in the United States are disproportionately lacking in communities of color and low income communities [42,43,44]. Urban transport and the uneven access to quality transportation is also a distributive environmental justice concern [44,45]. 

When it comes to the intersections between land use and environmental justice in the border region, there is ample knowledge about overall trends but little localized, street by street data. Border scholars have identified the lack of sewage and water infrastructure and the lack of urban tree cover in border cities [46,47,48]. More data on the type of land uses detrimental to border resident’s health will be critical for understanding the particular planning strategies that will be important for addressing environmental injustices in the region.

Procedural injustice is also at play in the land use planning process. Many facets of urban planning are characterized by expert-led processes where there is limited community participation in decision-making processes [45,49]. Traditional methods of community participation like townhalls and public comment have largely not proven to lead to inclusive public involvement [50]. Moreover, digital methods like Public Participation GIS have helped harness specific information to inform the planning strategies addressing environmental injustices [51,52]. Urban green infrastructure planning, for example, lacks “fine-scaled socio-perceptual information” to have a more integrated planning process with community participation, and PPGIS can generate this needed data [53]. Analyses of the impacts of PPGIS on the urban planning process have indicated that there are limitations to the ways in which community mapping informs land use decisions. While some argue that community mapping data is the most strategically used during the problem identification stage of the urban planning process, there is more need to have this data fit within the intervention stage [52,53,54]. While there are examples PPGIS used in addressing procedural justice in urban planning, there is still limited evidence pointing to its impacts on influencing the process for several reasons, including the lack of resources to support effective community participation [52,55] and resistance from planners [56]. This research aims to analyze the role that PPGIS plays in addressing border environmental justice through its impact on the land use planning process in Tijuana, México.

Another key actor in the urban planning process is community based organizations. These organizations participate in the land use planning process through advocacy. The role that PPGIS plays in building community organizing capacity is an important area of research. Participatory mapping helps community residents create a space for their perspectives in decision-making spaces where land use and environmental governance decisions are made [57,58]. Moreover, community mapping has been argued to empower community members by providing space for them to represent their issues visually [59,60]. The process of community mapping has been shown to generate an understanding of the planning process and this educational impact can have positive impacts on participation [61]. However, critics identify that not all organizations will be able to use the GIS technology and data in effective ways without the support of experts in the field [62]. This paper seeks to answer three research questions: (1) what are the key environmental risks and goods in the Zona Alamar? (2) what is community mapping project’s influence on the land use planning process? and (3) what are the mapping project’s impacts on community organizing capacity in the Colectivo and RECIMEC?

## 3. Materials and Methods 

**Figure 2:**
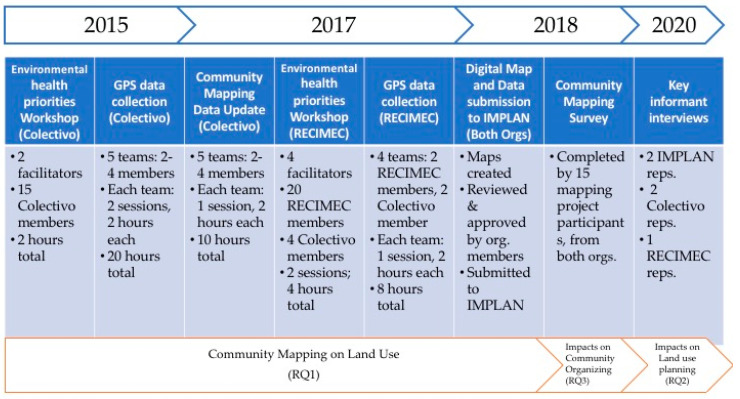
General community mapping workflow.

To answer these questions, a mixed method approach was employed that includes a community-based community mapping process, a survey, and five key informant interviews. The timeline and progress of these methods is outlined in Figure 2 below. 

The first research objective in this paper is to identify the type of land uses that are critical environmental ills and goods in the eastern region of Tijuana, México. The community mapping process included four phases: (1) priority identification, (2) mapping training, (3) data collection, and (4) data organization and map development. At the beginning of the community mapping process for each organization, a popular education-style brainstorming session was conducted to create a list of environmental justice issues that community residents identified as important in their community. First, the overarching themes around environmental risks and goods such as transportation, environmental health, pollution, and access to environmental goods like green spaces and schools, were created by the Colectivo organizer and the lead author before the workshop session. These themes were selected using the historical, localized expertise of environmental justice held by the two co-facilitators. Then, with these overarching themes on flip boards, organization members wrote down all the specific land uses and issues they experience in their communities. Members placed their different concerns like water runoff points, parking lots for semi-trucks, and recycling centers within these larger themes. This also included some environmental goods like libraries and parks. The co-facilitators went around reading out loud each of these specific issues and asked for participants to vote whether they perceived this issue impacted their health and well-being. After all the items had votes, participants decided together which items to keep as the most important and which to save for later based on which issues had the most votes. These mapping priorities became the sites to be identified through the community based mapping activities. While there are shared priorities identified in the Colectivo and RECIMEC work areas, there are important differences each of their work area’s regional context as seen in the results section. 

The second phase mapping training was organized by Colectivo organizers, and included an overview of the mapping project’s purpose, and a recap of the community priorities identified. There was a step-by-step training on how to use our GPS tracker, the Garmin eTrex Venture (Garmin International, Inc., Olathe, KS, USA). to collect data points and how to include supplementary information on our data spreadsheets. The data collected in the spreadsheets were the GPS coordinates, name of the business, type of establishment, and notes on any particular issues. Not all sites were businesses; some were sites like contaminated water runoff points, clandestine trash dumps, or trash burning sites. The instructions that team members received was to collect information on the sites identified as priorities in the first workshop. The only details other than the location and category of these sites that participants were asked to collect were any particular details that might help in directly intervening in the issue. For example, if a site was marked as a trash burning site, the notes might indicate that there was copper being burned or domestic waste. This information did not go into the GIS layers, but was documented for future complaints to city level authorities.

In order to collect the data required, the organizations created teams based on the colonias, or neighborhoods, everyone lived in. The Colectivo’s work area included the neighborhoods directly surrounding the Chilpancingo neighborhood, and the RECIMEC work area was in three eastern Tijuana neighborhoods; these regions were chosen as organization members held expertise in the areas. The Colectivo process had five teams with two to four Colectivo members along with the lead author who was the driver for all teams; there was a total of 15 Colectivo members who participated. A total of 325 usable data points were gathered within the three months. These data points were locations of critical environmental ills and goods identified as priorities to the community organization members. 

To facilitate the organization of the data collected, an excel database was created. Through this database, the data points were organized by community priority theme in order to provide a series of final results maps as an end product. These data points and maps were sent to IMPLAN for analysis and incorporation into the ZASP. The 2015 maps reflected the relationship between sites where people gathered and the sites where environmental ills were found. Though results were submitted to IMPLAN to be incorporated in the ZASP in 2015, a change in political administration influenced a complete halt to this project. Two years later, in September 2017, members of the Colectivo and IMPLAN met again to talk about the restart of the ZASP process that year. After this conversation, two decisions were made. First, the Colectivo decided that it was necessary to update the 2015 community mapping data, as three years can mean significant changes in a rapidly changing region like the Alamar. Sites that didn’t exist anymore were removed, and new sites of environmental risks or goods were added. A total of 261 data points were updated, with a third of these being new data points. Changes observed between 2015 and 2017 included the discontinuation of certain sites, like the clean-up of clandestine trash dumps due to new construction, or small businesses moving to other areas of the Colectivo’s work area. This updating process took nine sessions to complete with different Colectivo member teams, each session took about two hours. The Colectivo members who participated in the 2015 data collection also participated in the 2017 data collection. However, there were new members of the organization in 2017 and two of them participated in the community mapping process. 

Second, IMPLAN members suggested that the community mapping process expand to other areas of the ZASP planning area, especially to those areas closest to the forested region of the Alamar River, a key environmental asset in the region. The Colectivo saw this as an opportunity for collaboration with RECIMEC as they had already worked together in the process to protect the Alamar River. Starting in December 2017, the Colectivo, Carolina Prado, and IMPLAN representatives began the process of engaging RECIMEC members in a community mapping exercise to contribute their priorities and data points to the ZASP. The first phase in this mapping process was similar to the beginning stages of the Colectivo’s community mapping project. The first workshop was facilitated by IMPLAN’s members, Carolina Prado, and Colectivo mapping team members, and was focused on introducing the Zona Alamar Specific Plan to RECIMEC community members. The director of IMPLAN’s environmental division presented the basic elements of the ZASP. Colectivo members facilitated an activity to identify the types of priorities RECIMEC members were interested in having represented through their maps. At the end of the workshop, a schedule for the data collection process was created with members of three colonias: 10 de Mayo, Insurgentes, and Granjas Familiares. 

Beginning 18 January 2018, the RECIMEC went out throughout the three communities to gather data for this region’s contribution to the Zona Alamar Specific Plan. Data collection was collected by four teams with two RECIMEC members each and one Colectivo member; a total of 8 RECIMEC and 5 Colectivo members participated. Each group held one session to complete the street-by-street data collection and a session took about 2 h. At the end of this process, there were a total of 119 data points collected in the three communities. After the data collection was finished, this data was uploaded into ArcGIS (ESRI, Redlands, United States) and Photoshop (Adobe, Mountain View, United States) to create a series of maps to report back on. The data used in ArcGIS was the 2017 Colectivo and 2018 RECIMEC data. There was no spatial analysis of the data conducted on this software, other than an exploratory layering of 2010 Census Data that was not published. As community members don’t hold the knowledge and access to use ArcGIS, we decided to do a simple spatial analysis like this to do a rough estimate of a direct relationship that concerns many Colectivo members. There was a participatory spatial analysis of relationship between environmental health risks and schools done by two Colectivo members and the lead author, where rough estimates of distance radius were used based on the paper maps.

During a community workshop in March 2018, the results of the mapping data collection were presented to Colectivo and RECIMEC members. This session included an activity to identify the top seven priorities each organization wanted to highlight as the most critical to include in the ZASP. The last activity in the workshop was to identify action points for these community priorities, including actions that could be addressed by the Zona Alamar Specific Plan, and actions that could be taken through community organizing. This workshop revealed there were some data points missing in our maps, so data collection was wrapped up with two additional sessions in March 2018, one for the Colectivo and one for the RECIMEC area. Then, all the maps were updated with the new data. During a May 2018 workshop, the maps and results were reviewed and approved by both organizations. This workshop had 15 participants who were involved with the community mapping project and included presentation of the final ArcGIS maps. Results were organized by the themes presented in the original ZASP. The full report was printed and distributed to members of the Colectivo and RECIMEC during this workshop. After some minor feedback was implemented, the two organizations presented the community mapping project results formally to IMPLAN. 

In order to answer research question two, two of the five key informant interviews were conducted in 2020 with IMPLAN staff members who were actively working on the ZASP during the 2015–2020 timeframe. One of the IMPLAN members was the director of the environmental sector of the urban planning agency and was in charge of the community outreach for the ZASP process. The second representative is the director of the metropolitan urban plan. The semi-structured key informant interviews were conducted by phone, included seven questions, and lasted about one hour. The recordings were transcribed, translated, and analyzed using qualitative coding in NVivo with a “scissor and sort” technique [63]. Further research to answer this question in the future will include a content analysis of the published ZASP.

To analyze the impacts of the community mapping process on the organizing capacity of organization members, a short survey was conducted during the May 2018 workshop. The questionnaire prompted participants to agree or disagree with statements such as “I feel safe in my ability to talk with my neighbors about injustices in my community,” and “I think that this project will contribute to building better community.” The write-in sections of the survey included three prompts: (1) Why did you decide to participate in the mapping project? (2) What do you like most about your participation in the project? and (3) What do you think could have changed in the organization of the project? Fifteen participants filled out the survey out of twenty-three participants from both the Colectivo and RECIMEC (65% of participants). No identifying information was collected in the survey. While there isn’t a precise breakdown on how many members of each organization participated, workshop participation included about 70% Colectivo members and 30% RECIMEC members present. Fifteen participants, made up of both Colectivo and RECIMEC members, answered a short 10-question survey. This survey included a Likert scale questionnaire with 10 prompts and three write-in sections. This data was supplemented by a small focus group conducted with Colectivo and RECIMEC representatives in November 2020 on the question of mapping project impacts on community organizing. 

## 4. Results

In this section, the environmental health issues identified by the partner organizations and the results of the data collection process on these issues are presented. Second, the community mapping project results’ influence on the urban planning process and on community organizing capacity in the organizations’ membership are discussed. 

### 4.1. Community Environmental Health Priorities 

**Figure 3:**
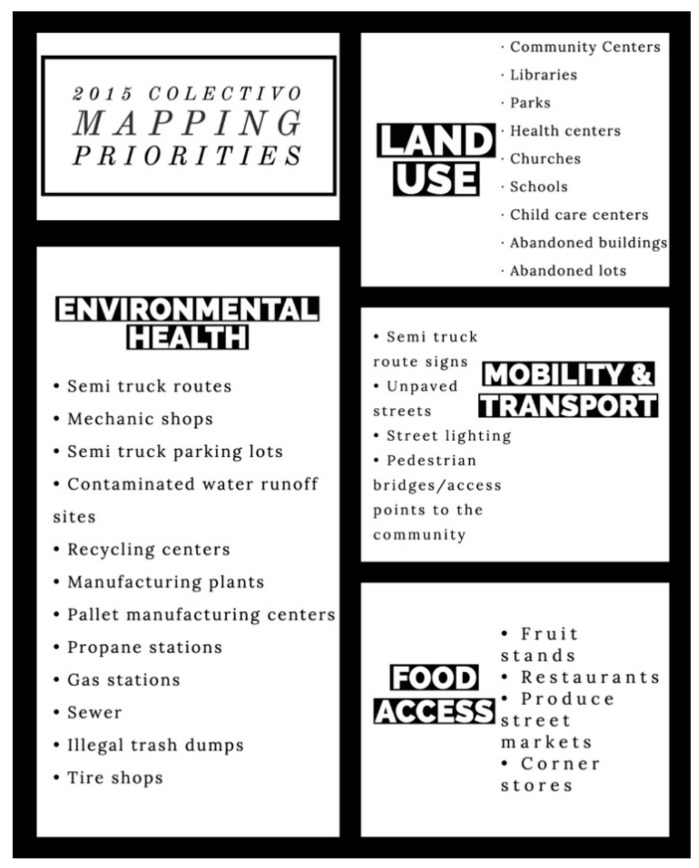
2015 Colectivo mapping priorities.

The Colectivo’s work area is a region of the city that hosts a great deal of industrial activity. Therefore, the biggest area of concern for this community was the impact that manufacturing plants and their related activities have on their community. As seen in Figure 3, key issues include services that support semi-truck traffic like parking lots, pallet manufacturing centers, and mechanic shops. Moreover, recycling centers, manufacturing plants, and contaminated water runoff sites were critical issues as well. 

**Figure 4:**
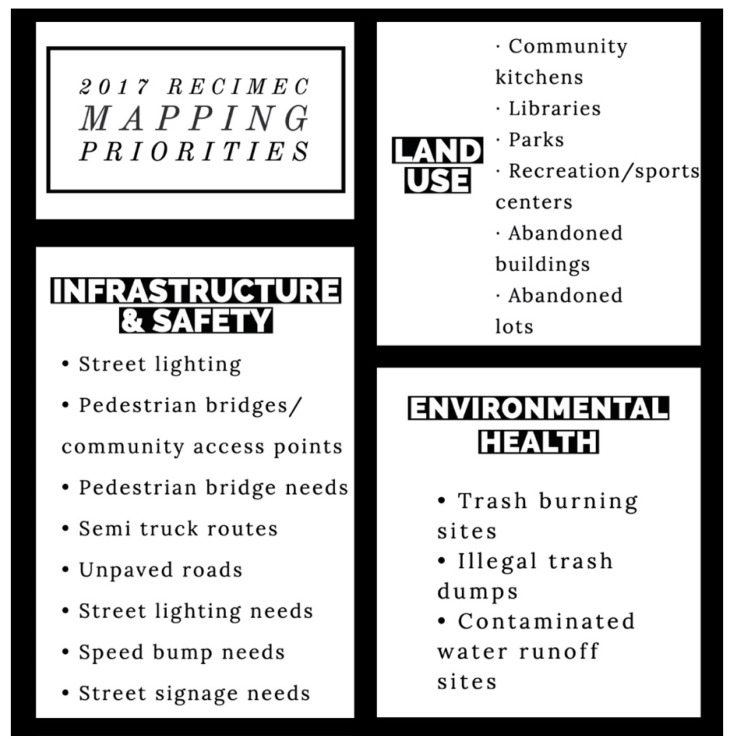
2017 Red de Ciudadanos por el Mejoramiento de las Comunidades (RECIMEC) mapping priorities.

In contrast to the Colectivo, RECIMEC’s work area is a bit more undeveloped with substantial infrastructure needs. While the community members did have concerns that are specific to environmental health, they also focused more on infrastructural issues that can benefit public safety. The environmental health risks, reflected in Figure 4, are also related to infrastructure issues such as the irregular garbage pickup in this region of the city. Residents also identified the lack of social services and abundant abandoned lots as impactful to their health and well-being.

In the subsection of the Zona Alamar where street-by-street data was gathered, there were important environmental health hazards. The main issues in the Zona Alamar are contaminated water runoff points, lack of public infrastructure, abandoned lots, clandestine trash dumps, industrial plants, recycling plants, and heavy truck traffic that is influenced by businesses such as semi-truck parking lots, pallet manufacturing centers, and semi-truck mechanic shops. Twenty contaminated water runoff points that residents are exposed to in residential areas, school zones, and public roadways. Industrial activity has an impact on environmental health in the region: 12 recycling centers and 43 manufacturing plants were identified in the region.

**Figure 5:**
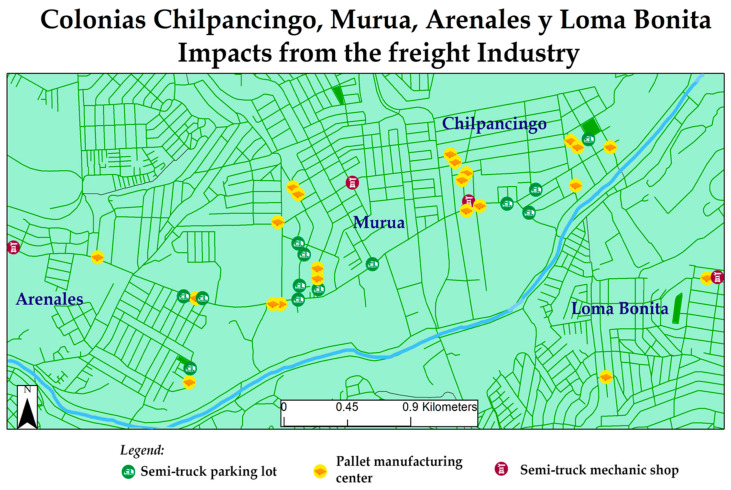
Colectivo region freight industry impacts.

Particularly in the Colectivo’s work area, there are impacts from semi-truck traffic (see Figure 5). Pallet manufacturing centers and semi-truck mechanic shops are businesses that promote semi-truck traffic in the region; there are 28 pallet manufacturing centers and 5 semi-truck mechanic shops in the area. There are 19 semi-truck parking lots that also become a safety and health risk as this promotes more semi-truck traffic in the residential areas, near schools.

**Figure 6:**
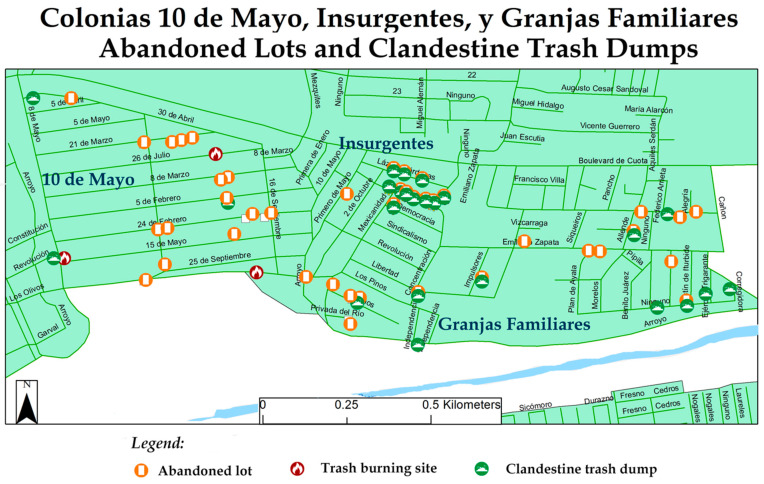
RECIMEC region abandoned lots and trash dumps.

Infrastructure and environmental health risks are closely intertwined in the Alamar region (see Figure 6). The lack of regular garbage pickup creates important issues including 74 clandestine trash dumps and six trash burning areas, specifically in the RECIMEC work area where there are more issues with illegal burning. Out of all the trash dumps, 72 percent of these are in abandoned lots. There are 137 abandoned lots in the Zona Alamar that become a public safety and health risk, as they become sites for trash dumping and/or illegal activities. Moreover, the Zona Alamar is in need of public infrastructure that supports social services. As of the last mapping efforts, there are zero libraries, and only eight parks for a 2010 population of 39,217.

While further vulnerability analysis is needed, a collective analysis of the relationship between environmental health risks and schools affirmed the concerns of many of the organization’s members. Through a workshop activity, many environmental health risks were identified close to schools, especially clandestine trash dumps, welding shops, and pallet manufacturing plants. All schools in the region analyzed had at least one of these environmental health risk sites within an 800 foot (1/4 km) radius. Moreover, more than half (61%) of schools in the mapping area were located within a radius of at least ten environmental health risk sites. Further systematic analysis is needed to determine more areas of vulnerability in the region. 

### 4.2. Influence on Community Specific Plan Process

The process of this report’s uptake from IMPLAN is nonlinear, as the political administration has vastly changed current mandate of the urban planning process in Tijuana. Right before the community mapping project report was submitted to IMPLAN, there was a change in administration in December 2016. Therefore, while the information provided to IMPLAN was reviewed upon submission, the ZASP was de-prioritized in the institution’s timeline. The new administration brought with it a new priority—the Urban Development Program for the Central Population, or the acronym PDU CPT in Spanish (used henceforth). This plan covers the metropolitan area encompassing the cities of Tijuana, Tecate, and Rosarito. It is a macro-level urban plan that is slated for completion in 2021. The current IMPLAN administration has this PDU CPT as a priority, and from there aims to work on the community-specific plans, like the ZASP. Key informants for this project indicated that it is the most likely that the ZASP will be revisited between 2021–2025. The influence of this report on the urban planning process was (1) ground truthing the land use data that IMPLAN held for the Alamar region, (2) informing the diagnostic analysis of the metropolitan urban plan, and (3) highlighting the urgency of implementing the ZASP in a timely manner.

While it is a setback for the community mapping project’s goals to have the ZASP pushed back in such a significant way, the project’s findings have still impacted the urban planning process. The primary impact of the project, according to the two IMPLAN representatives interviewed, is that the project provided an important “ground truthing” of the land use information already held by IMPLAN. The former director of the Department on Environmental Issues at the institute indicated that the geolocated data provided by the mapping project helped IMPLAN staff verify the precision of their information and update the land use maps they had already created. Supporting this argument, the Director of Urban and Territorial Planning at IMPLAN indicated that the contribution of this project is especially imperative as the institute is usually lacking in staff and funding to be able to do the detailed field work required to verify land use information gathered from satellite data. Therefore, the community mapping data was critical to gathering accurate and timely data on land use and activities in the region, especially those that are impacting the local community. 

Though the information on specific community priorities and recommendations for urban planning strategies have yet to be incorporated in the community-specific plan for the Alamar region, they are currently being incorporated in the PDU CPT. This incorporation in the metropolitan urban plan has two key functions: (1) it creates a link between the macro-level goals of the metropolitan urban plan and the localized needs of the Alamar region, and (2) it highlights the importance of revisiting the ZASP in a timely manner after the PDU CPT is written into law. Informing the diagnostic section of the metropolitan urban plan allows there to be an important link between regional and community-specific priorities. The Director of Urban and Territorial Planning said, “We have incorporated some of the key priorities, around public infrastructure and clandestine trash dumps, specifically, to the PDU CPT to be able to interlink this information to the macro. This way when we restart work on the community-specific plans we are able to pay attention to these identified problems.” Moreover, this director indicated that while placing these community-specific issues in the metropolitan plan doesn’t legally mandate that they be addressed in a timely manner, it does give them a bigger importance in IMPLAN’s mandate. She said, “the fact that these issues show up in such a specific way in the PDU means that they have a critical importance when it comes to the environment […] it increases the issues’ importance.” This strategic incorporation in the metropolitan plan is important for shedding light on two critical environmental problems gleaned by IMPLAN representatives: lack of public infrastructure and clandestine waste dumping.

The third influence that the community mapping project findings have on the urban planning process is to indicate the time sensitive nature of implementing a community- specific plan for the Alamar region. The PDU CPT will have an impact on the community-specific plans that will be revisited in the coming five years. Incorporating the community mapping data has highlighted key environmental priorities and has shown the importance of creating a community-specific urban plan in the Alamar region. The Director of Urban and Territorial Planning indicated “If the issues in the Alamar region are not represented in the metropolitan plan, it is complicated to address it in our institutional calendar. Incorporating this information allows us to address the priorities in a shorter time frame, sometime between 2021–2015.” 

### 4.3. Impacts on Community Organizing Capacity

The mapping project had three impacts on the community organization capacities of the two organizations. First, organization members were able to better identify community issues and solutions. Second, the project helped catapult action on two community issues as well as served as a consciousness raising strategy for improving community members’ individual environmental behavior. Last, the mapping project served as a mechanism for creating inter-organization connections between the Colectivo and RECIMEC.

**Figure 7:**
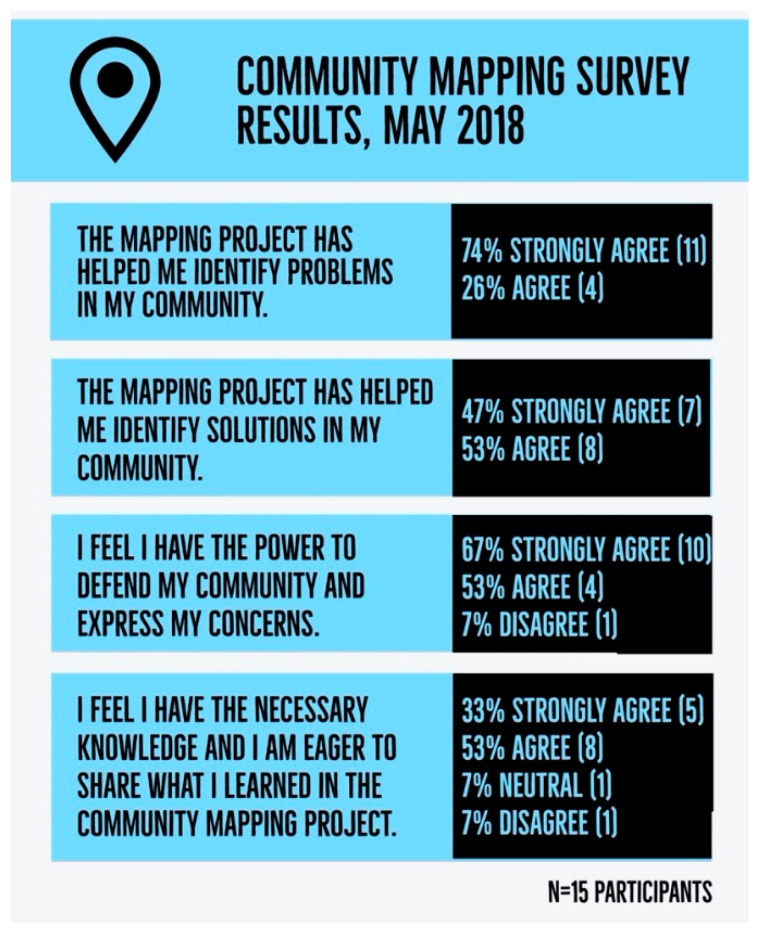
Community mapping survey results, May 2018.

As organizations, both the Colectivo and RECIMEC aim to build a base of organized community members to tackle neighborhood issues. One of the goals for the community mapping project, in addition to influencing the outcomes of the urban planning process, was to increase the community organizing capacity of both organizations. The community mapping results were largely positive from participants who indicated that they felt that the project had been helpful for them (for key findings, see Figure 7). 

When asked what the most meaningful part of their participation in the community mapping project was, eleven out of the fifteen participants identified networking and collaboration as the key elements. The community mapping process was an exercise in inter-organization collaboration on environmental justice, especially between longer-term activists and residents that had less experience with community organizing. One organization member said she appreciated that “all of us in the group are united, we are all participating, and are able to help our community.” Another participant noted that getting to know other community members was very important in her learning process. This mapping project was able to influence solutions on the environmental justice priorities identified that are relatively modest, but the overall impact on inter-organization capacity was strong. Colectivo and RECIMEC members have expressed that the mapping project helped build trust between the two organizations’ base and this trust has been expressed by continuing to invite the other members to each other’s workshops and events. The connections that the project helped form between the two communities will be important for continued collaboration on environmental justice movement goals.

## 5. Discussion

The first research objective was to find the environmental risks and goods prioritized by community members in this border region. Some of the sites found are aligned with historical environmental justice issues identified in the U.S. [64] that identify localized risks like truck traffic, auto mechanic shops, and noxious facilities as key issues. Global environmental justice analyses have identified other types of risks like resource extraction, transport and waste disposal [65], limited access to resources on conservation reserves [66], and access to safe drinking water [67]. On top of these expected indicators, though, the specificity of the border region has created particular environmental health risks. For example, pallet manufacturing plants aren’t necessarily creating hazardous conditions for community members on their own, but their location within residential areas does. Since semi-trucks pick up pallets for their freight trips, these pallet manufacturing plants create air quality issues for residents. Another key priority that is influenced by the specificities of the border region is that of clandestine trash dumps and abandoned lots. The city of Tijuana, like other industrial border cities, faced rapid development, and many areas have experienced different waves of construction because of factors like external and internal migration. This has left regions of the city like Colonia 10 de Mayo with many abandoned lots that become sites of clandestine trash dumps. These dumps expose residents to household and industrial waste. 

The environmental health priorities identified by the community mapping project are important for expanding the understanding of localized border environmental justice experiences [9,68]. Highlighting the kinds of experiences local residents see impact their environment and their health is important for identifying future research and advocacy campaigns. Findings from this project contribute to the field of global environmental justice by pointing to the kinds of activities and risks that have confluence with other environmental justice movements and the issues that are specific to the U.S.-México border region. 

Analyzing the impact of the community mapping project on the land use planning process was the second research objective. Results from this evaluation align with some of the existing dynamics within the field of Public Participation GIS. A key role PPGIS plays is that community members use their local knowledge to inform valuation and understanding of particular places and the conditions they influence [21,22]. Public Participation GIS has helped harness specific information to inform the planning strategies that are used to address environmental injustices [50,51]. IMPLAN representatives indicated that the community mapping results have informed the diagnosis process of the city-wide urban plan. The environmental health risk data served as ground truthing for the limited data that the agency had on the eastern region of the city. This data informed the critical issues that the city-wide urban plan must outline as priorities for urban development and zoning. 

Though there are significant impacts on the land use planning process, there are some limitations to this influence. First, community mapping data is often used in the problem identification stage of the urban planning process and less so in the intervention stage [52,53,54]. While IMPLAN representatives did allude to some influence the project had in creating planning interventions for the Alamar region, to date there are no concrete examples of this in action. Second, while the impact on the land use decisions will be used in the metropolitan urban plan, these might not lead to the shorter-term changes that organization members were expecting from the local-level plan. Both IMPLAN representatives indicated the urgency of these issues, and how the urban planning process is not necessarily aligned with the processes going on in the communities most impacted. This is a key limitation to the PPGIS approach, as participation in governance processes like urban planning might seem inefficient in light of the severity of community issues.

Participatory mapping helps community residents create a space for their perspectives in decision-making spaces where land use and environmental governance decisions are made [57,58]. In order to be able to participate in the land use planning process, community organizations need to build capacity. The third research objective is understanding the impacts of the community mapping project on community capacity and organizing. This project’s key influences were to build coalition between the Colectivo and RECIMEC as well as creating capacity for these organization members to participate in the urban planning process. Having horizontal collaboration between environmental justice organizations within border cities is critical for building a common advocacy base. Through the community mapping project, residents from both organizations got to work together in teams and understand each other’s community priorities better. Interactions with IMPLAN representatives helped organization members understand the urban planning process and to identify how they can intervene within it, an output identified from other community projects [61]. One critical impact this project had on community organizing was on fomenting stronger relationships between Colectivo, RECIMEC, and IMPLAN members. To date, Colectivo and RECIMEC organization members continue to have an open line of communication with IMPLAN representatives around urban development and zoning in their region. This type of relationship building is critical for being able to reach the two organizations’ policy advocacy goals. Some limitations to PPGIS projects are the ways in which GIS technology is not accessible to many community members [62]. This limitation was definitely present in this project as the mapping required a “PPGIS facilitator” [69] to create the final maps on ArcGIS.

An interesting finding on community organizing capacity is that while a majority of organization members identified that the community mapping process supported them in identifying problems in their community, less members were in complete agreement with the statement that the project had helped them find solutions. However, there were at least two key solutions that were enacted as a result of the community mapping project. First, the Colectivo was able to begin their campaign to identify sources and impacts of water contamination sites. This had been an active concern of organization members for years, but the mapping project provided data points to initiate a systematic investigation on where the polluted water is coming from and identifying potential chemical agents. Second, a small group of RECIMEC members got together to submit a formal petition requesting service on street lighting posts in their community. These community members used the data gathered from the project to identify at least eight street lighting posts that had been faulty and to request that the city service them. In addition to these action points, the mapping project served as a strategy of consciousness-raising for the community members to understand the impact of their personal behavior on the conditions of their neighborhoods. Colectivo members expressed that performing the on-the-ground research this project required helped them see their own role in contributing to community issues, especially with issues like trash burning.

While there are important contributions identified, this community mapping project also faced substantial limitations. First, to address the question of PPGIS impacts on the land use planning process, the number of people interviewed was limited. Though the interviewees have key expertise in this process, it will be worthwhile to analyze this impact through comparison between the published urban plan and the ZASP draft the community mapping project used as a reference. Moreover, future research should include more IMPLAN interviewees when the ZASP is worked on actively again. Second, the impact on community organizing was based in a survey with about two thirds of the participants and three key informant interviews with community organization members. In the future, it will be useful to have more qualitative data to work from like a focus group with organization members who participated in the project. Unfortunately, the COVID-19 pandemic impeded this opportunity, but a group conversation on this topic will be critical for understanding community organizing impacts. 

## 6. Conclusions

Our key findings point to particular environmental challenges in this border city including clandestine trash dumps, and an important contribution to the city-wide urban plan through problem identification and ground truthing. The community mapping project also had a key impact on community organizing through the fomenting of knowledge and relationships between community members and government representatives at the city’s urban planning agency. Through this project, Colectivo and RECIMEC members were able to foment capacity and confidence to participate in the struggle for the collective well-being of their respective environmental justice communities of the Alamar region. 

This data informs important policy recommendations for the ZASP process. First, for this particular community mapping project, a data update is recommended. The rapid urban development faced by industrial cities like Tijuana means that urban dynamics are rapidly changing. Even within the two year gap between the 2015 and 2017 data collection, there was a significant change in land use. Therefore, a recommendation for this urban planning process is to fund and support a community mapping project update every few years, and definitely at least once before the ZASP publication. Second, the Zona Alamar Specific Plan includes a wider area than the two organizations involved have the capacity to work on. There are still other neighborhoods to engage with on street-level data collection and priority identification. A key recommendation proposed here is to expand the data to include other communities in the region; the La Torres neighborhood next to the colonia 10 de Mayo would be an especially important addition. From this community mapping project process, issues identified were a high impact of freight, and very few schools in that colonia. The Colectivo and RECIMEC members could train community folks from these colonias to expand the community mapping project. 

Community based mapping projects like this should be supported by local land use planning agencies through funding and offering access/transparency to preliminary data. Having the ability to understand the kinds of issues already being addressed will be an important starting point for community organizations to plan their mapping projects. Urban planning agencies can support this public participation by offering capacity building in GIS. Environmental justice GIS researchers could also use research grant funding to work on this capacity building as well. This will help bridge the access gap with this technology and reduce the need for ”PPGIS facilitators/intermediaries.” Another way to improve this PPGIS approach is to build in an evaluation of the mapping project’s impact into the land use planning process into the study’s design. Creating formal collaborations between community-based organizations, like the Colectivo and RECIMEC, is important in building this long-term accountability and being able to evaluate the specific ways in which community mapping data creates changes in land use decisions. Last, environmental justice advocates can further influence the environmental risks and goods found by their community mapping projects by pursuing organized campaigns to address particular issues. 

## Data Availability

Data sharing is not applicable to this article.
